# Recommendations for implementing digital alcohol interventions in primary care: lessons learned from a Norwegian feasibility study

**DOI:** 10.3389/frhs.2024.1343568

**Published:** 2024-10-11

**Authors:** Sebastian Potthoff, Håvar Brendryen, Haris Bosnic, Anne Lill Mjølhus Njå, Tracy Finch, Torgeir Gilje Lid

**Affiliations:** ^1^ Centre for Alcohol and Drug Research, Stavanger University Hospital, Stavanger, Norway; ^2^ Department of Social Work, Education and Community Wellbeing, Northumbria University, Newcastle upon Tyne, United Kingdom; ^3^ Department of Psychology, Faculty of Social Sciences, University of Oslo, Oslo, Norway; ^4^ Norwegian Reading Centre, Faculty of Arts and Education, University of Stavanger, Stavanger, Norway; ^5^ Department of Nursing, Midwifery and Health, Northumbria University, Newcastle upon Tyne, United Kingdom; ^6^ Faculty of Health Sciences, University of Stavanger, Stavanger, Norway; ^7^ Research Unit for General Practice, NORCE Norwegian Research Centre, Bergen, Norway

**Keywords:** risky drinking, hazardous drinking, alcohol, digital interventions, eHealth, implementation science, normalization process theory, primary care

## Abstract

**Introduction:**

Excessive alcohol consumption is a leading global risk factor for ill-health and premature death. Digital alcohol interventions can be effective at reducing alcohol consumption, but their widespread adoption is lagging behind. This study aimed to identify factors promoting or inhibiting the implementation of a digital alcohol intervention in Norwegian primary care, by using Normalization Process Theory (NPT).

**Methods:**

A mixed methods feasibility study combining quantitative and qualitative methods. A digital alcohol intervention called “Endre” was implemented across four GP practices in Stavanger and Oslo. Usage of the intervention was logged on the digital platform. General practitioners (GPs) reported their perceived uptake of the intervention via a web-based survey. The Normalization MeAsure Development (NoMAD) survey was used to measure support staff's perceived normalization of the intervention. Qualitative data were analyzed using the NPT framework, with quantitative data analyzed descriptively and using *χ*^2^ and Wilcoxon signed-rank test for differences in current and future normalization.

**Results:**

Thirty-seven GPs worked in the clinics and could recruit patients for the digital intervention. Thirty-six patients registered for the intervention. Nine patients dropped out early and 25 completed the intervention as intended. Low normalization scores at follow-up (*n* = 27) indicated that Endre did not become fully embedded in and across practices. Nonetheless, staff felt somewhat confident about their use of Endre and thought it may become a more integral part of their work in the future. Findings from six semi-structured group interviews suggested that limited implementation success may have been due to a lack of tailored implementation support, staff's lack of involvement, their diminished trust in Endre, and a lack of feedback on intervention usage. The outbreak of the Covid-19 pandemic further limited opportunities for GPs to use Endre.

**Conclusion:**

This study investigated the real-world challenges of implementing a digital alcohol intervention in routine clinical practice. Future research should involve support staff in both the development and implementation of digital solutions to maximize compatibility with professional workflows and needs. Integration of digital solutions may further be improved by including features such as dashboards that enable clinicians to access and monitor patient progress and self-reported outcomes.

## 1 Introduction

Excessive alcohol consumption is linked to a range of detrimental health, social, and economic consequences, making it a major global health risk (1). In Norway, registered alcohol sales between 2010–2019 ranged from 5.97 to 6.59 L pure alcohol per person 15 years or older, but surpassed 7 L of pure alcohol per person in 2021, during the covid-19 pandemic (2). Thirty-six percent of the Norwegian population reported drinking on a weekly basis and 50% engaged in heavy drinking within the past year (2).

Alcohol screening and behavioural interventions implemented in primary care settings have shown promise in reducing alcohol consumption (3–5). These interventions have the potential to reach a large proportion of the population at a relatively low cost (6). By leveraging digital platforms, such as smartphone apps, primary healthcare providers can offer patients timely support outside of consultations. These digital interventions often employ theory-based techniques to assist patients in monitoring their alcohol intake, enhancing self-regulation, and avoiding relapses during critical moments (7). Some interventions have features that facilitate integration into healthcare, such as dashboards that enable clinicians to access and monitor patient progress and self-reported outcomes (8, 9).

Meta-analyses have provided evidence demonstrating the effectiveness of digital interventions in reducing alcohol consumption (10, 11). Numerous evidence-based apps have been designed specifically to support individuals in decreasing their alcohol use (12–14). However, despite their proven efficacy, the implementation of digital alcohol interventions in primary care systems still lags behind (15). Multiple barriers hinder the widespread adoption of digital interventions in primary care, including individual (practitioner and patient), organizational (practice), and broader health system factors (16, 17). For instance, the implementation of digital interventions will likely require healthcare teams to undertake new responsibilities, such as introducing patients to the technology, monitoring their usage, and providing support and guidance throughout the process (18).

Given these challenges, rigorous research is necessary to uncover the factors that shape the implementation of digital alcohol interventions and identify the processes within clinical care that facilitate their delivery (19, 20). The application of implementation science theories, models, and frameworks can support this effort by connecting insights from individual studies to the broader evidence base, thereby informing effective intervention strategies and promoting knowledge translation (21, 22). In fact, the Medical Research Council framework for developing and evaluating complex interventions recommends early consideration of implementation, including during the feasibility testing stage (23). As such, the current study aimed to investigate the implementation of a digital alcohol intervention called “Endre” for at-risk drinkers in Norwegian primary care. This feasibility study addressed two important questions: (1) what is the uptake of the intervention in clinical practice? and (2) what are primary healthcare providers’ perceived barriers and facilitators regarding the implementation of the intervention? The findings from this study will inform recommendations for more effective implementation of digital alcohol interventions in primary care.

## 2 Materials and methods

### 2.1 Design

This feasibility study incorporated mixed methods (semi-structured group interviews combined with quantitative surveys) to investigate the implementation of a digital alcohol intervention in Norwegian primary care (24). The digital intervention was designed as a supplement to the GP's follow-up of the patient, and it was introduced to the GPs embedded in a seminar series on pragmatic case finding and management of alcohol-related health problems delivered in their clinics (25, 26). In this study we drew on the Normalization Process Theory (NPT), an internationally recognized framework for understanding how individuals work together to integrate and embed new practices into routine care (27). Normalization is proposed to occur via four generative mechanisms: “coherence”: how people make sense of what needs to be done, “cognitive participation”: how people get involved and take responsibility for the new intervention, “collective action”: how people work together to make practices work and “reflexive monitoring”: how people assess the impact of the new intervention. The NPT has been widely used in the past to investigate the implementation of brief alcohol interventions in primary care settings in other countries (20, 22).

### 2.2 The digital intervention

The intervention was originally developed as a smoking cessation program and its development is described in detail elsewhere (28). It is a web-application designed to be easily accessed and read on small screens, like smart phones. The current version of the program is a theory-based, self-administered e-health intervention to address behavior change and alcohol-related health problems for use in general practice, and it consists of 22 unique modules, which take between 3 and 10 min each to complete. The theoretical basis is mostly drawn from Self Determination Theory (29), Motivational Interviewing (30), and relapse prevention (31). The main focus of the program is to support behavior change through computerized motivational interviewing (32) to strengthen internalized motivation, and the program is designed to support a working alliance between user and program (33–37). The intervention thus uses a virtual partner (i.e., a non-embodied relational agent) called “Endre” (a male first name in Norwegian, but also Norwegian for “change”, and the name of the intervention). Endre uses first person tense, asks questions and answers empathically, uses humor, greetings and farewells, “remembers” earlier conversations by referring to them or adjusting program content in accordance with them, and several other alliance supporting elements. The intervention also entails a component designed to raise patients’ awareness of how their alcohol use may relate to their health problems. As such Endre is intended as a supplement to how GPs manage the follow-up with patients. This relates to both the treatment of the health problem they visited their doctor for, and the follow-up of a possible alcohol problem, be it follow-up appointments with the GP, referral to other services or medication. The intervention is supposed to be used by patients in their homes, or wherever they roam. Inactive users received up to three reminders on the web-service/Endre, first by SMS after 2 days, then by email after 1 week, and finally by email after a fortnight of inactivity (non-usage/not logging on to the web-service). Screenshots of the intervention can be found in the Supplementary Figures 1, 2.

### 2.3 The staff training

We applied the Behavior Change Wheel approach to develop a tailored intervention to implement Endre in clinical practice (26, 38). This involved the use of the Capability, Opportunity, Motivation-Behavior (COM-B) model to explore the determinants of GPs’ behavior in context. The final implementation intervention consisted of four seminar sessions with 4–8 weeks interval (3–4 h each). Four sessions were chosen to enable GPs to fulfill the Norwegian requirement for qualification and requalification, which includes at least five clinical seminars of at least 15 h every 5 year period. All sessions included approximately 30 min on Endre. Due to technical issues, two clinics started using Endre after the first session, and two after the second session. Importantly, the training in Endre was only a secondary aim of the seminar series and only took up a small part in the sessions. The main aim of the seminar series was to equip GPs and support staff with the skills and tools to deliver a targeted approach to addressing alcohol in general practice (26). This approach is based on clinical relevance, meaning the practitioner addresses alcohol when it is potentially relevant to the condition that the patient is presenting with, either as cause, complicating factor or due to increased vulnerability (25). Training activities were tailored to address professionals’ prospectively identified needs with regards to delivering targeted alcohol interventions in primary care. This included tools for screening for hazardous alcohol consumption, motivational interviewing techniques and techniques for following-up and maintaining change. Endre was provided specifically to address GPs’ need for an accessible electronic resource which could be used by patients between sessions to support reduction of their alcohol consumption. The systematic development of the seminar series is reported elsewhere (26).

All clinics received a link to a functioning test version of Endre inviting them to test themselves before recruitment of patients started. Endre training was delivered by an e-health expert (HB) during the first 30 min of each of the four seminar sessions. An initial introduction addressed the technical setup of Endre, including instructions on how to support users with creating an account. The use of Endre involved GPs identifying eligible patients who had alcohol related health problems or for other reasons consented to using Endre and participating in the study. The initial plan was to let the GPs decide whether to include the patients themselves or leave it to support staff, but the regional ethics committee demanded that the signed consent form should not be received by the GP. Consequently, the GPs could not introduce the patients to the platform and help them create a user account. After feedback from two experienced GPs and support staff at two clinics, we decided that GPs should refer eligible patients to their support staff who would take the participant consent, introduce the digital platform and help them create a user account. This is in line with GPs ordering other services from their laboratory (e.g., blood samples, electrocardiograms). GPs identified eligible patients using the pragmatic case findings approach. This involved asking patients about their alcohol consumption when it was clinically relevant. GPs were provided with a list of clinical relevance which included common conditions with robust evidence of potential relevance for alcohol, where cutting down or stopping may likely improve the patient's current health status. The GPs were prompted to always think about alcohol for these conditions, and to offer Endre if the patient accepted that alcohol might be a factor for them. In addition, they should offer Endre in all other situations where the GP and/or the patient thought that alcohol consumption might play a part in their health problem, including for patients where this was already known. The following seminar sessions (2–4) involved the delivery of tailored training, troubleshooting and opportunities for staff to ask questions. In addition, there was on-demand technical support via phone and email. Waiting room prompts (small posters) advertising the project and the app encouraged patients to speak to their GP about alcohol and health problems.

### 2.4 Sampling and recruitment

We invited three GP practices in Stavanger and one in Oslo to participate in this feasibility study. This included three large practices (at least eight GPs) and one medium-sized practice (around five GPs). All four practices accepted the invitation to participate. Two of the practices had previously provided their perspectives in a needs assessment to tailor the clinical seminar series (26). Three of the practices were self-owned, and one was owned by the municipality. In total, 37 GPs and 22 support staff members participated in the seminar series at least once, and 24 and 14 respectively participated in at least three sessions. Participants for the semi-structured group interviews were purposefully sampled at the end of the seminar series to represent both the different roles (GPs and support staff), and the groups were arranged according to role. All support staff involved in the delivery of Endre were invited to complete the survey. Group interview and survey participants were recruited through direct contact with TGL who was responsible for coordinating the data collection for the clinical seminar series.

### 2.5 Data collection

#### 2.5.1 Actual usage of the digital intervention and recruitment source

Any use of the digital intervention was automatically logged throughout the study period. These data include the number of patients that completed registration, how many modules each patient completed, and at which center the patient was recruited from (unique recruitment link for each center). Upon completing the registration process the patients were asked which physician referred them to the intervention.

#### 2.5.2 Inclusion and dropout survey

When the recruitment of patients was ended, all GPs received an e-mail with a link to a web-survey. Non-responders received up to two reminder e-mails, and finally a paper and pencil version to their staff mailbox at the clinic. In this survey GPs were asked two questions about the recruitment of patients to Endre:

1. How many of your patients gave the impression, during the consultation, that they intended to participate in the study and use Endre?
2. What reasons for non-participation did the patients give? (i.e., those that explicitly rejected participation during consultation). Six categories were given: disagree that alcohol is part of the problem; do not wish to participate in research; do not have smartphone, tablet or pc; do not want to use a digital treatment solution; do not want to use Endre (for other reasons); and do not know/remember. GP were asked to give a number for each category.

#### 2.5.3 Group interviews

The first group interview was conducted by SP (PhD). All remaining group interviews were conducted by ALMN (MSc) with participants in their place of work using a theory-informed topic guide that evolved over time (39). Interviews were conducted between 27th November 2020 and 1st December 2021 and lasted an average of 28 min (range 21–38 min). Participants were interviewed in small groups to allow them to reflect and build on their colleagues’ perspectives, and the interviewer was present or communicating via video link. The interview topic guides for the GP and support staff interviews can be found in Supplementary Files 1, 2.

#### 2.5.4 NoMAD survey

We used the Normalization MeAsure Development (NoMAD) survey, which is a validated 23-item survey for assessing implementation processes from the perspective of professionals involved in the work of implementing complex interventions in healthcare (40, 41). We adapted the original version to assess the integration of the digital alcohol intervention in Norwegian general practice. Following a published translation protocol, the adapted English version of the NoMAD was translated into Norwegian using a forward-backward translation process carried out by independent translators (42). Discrepancies between the original English version and the back translated English version were analyzed with one of the original survey developers (TF). Changes were then integrated into the final Norwegian language version (Supplementary File 3). All staff involved in the delivery of Endre were asked to complete the survey after they had used Endre for some weeks (baseline, at the second or third session) and after the last seminar session to explore the degree of normalization of the digital intervention early on after the introduction, and after having used Endre for some time. Participants responded to NPT construct items using a 5-point scale of agreement for response (1 = strongly agree to 5 = strongly disagree). They also responded to three general “normalization” assessment items using an 11-point scale (0–10) with higher scores indicating greater levels of normalization.

### 2.6 Data management

All data were saved on the secure server for research data at Stavanger University Hospital, only accessible by designated persons. Group interviews were audio-recorded with participants’ consent and transcribed verbatim for analysis. All data were carefully anonymized to prevent identification of either the individual participant or the participating study site. Qualitative software (NVivo) was used to support data management, analysis and documentation.

### 2.7 Data analysis

#### 2.7.1 Qualitative data analysis

Qualitative data analyses were informed by NPT as a theoretical framework. SP led the data analysis, which was carried out in parallel with data collection and followed a two-part process. The first part involved an inductive thematic approach (43). This involved the inductive coding (using QSR NVivo 12) of three early group interviews to identify features, patterns, groups of codes and potential themes to build a preliminary framework for application to the data and to inform further data collection. These three coded transcripts were independently read by TGL and further discussed with ALMN. Next, analyses moved to a template approach which involved developing a coding template based on these discussions and applied to four group interviews (44). Further discussions of the transcripts, codes and themes were undertaken by SP, HB, and TGL who reworked the coding template as a result. Further development of the coding framework into themes was undertaken with all authors, consensus reached, and the codes and themes applied to the full data set. The themes were then mapped, integrated and interpreted alongside the four NPT constructs.

#### 2.7.2 Quantitative data analysis

R (version 4.2.2) was used for quantitative analysis. The data had some missing values, and these were imputed with a kNN regression algorithm. Survey items relating to the four NPT mechanisms were analyzed by examining descriptive statistics for each of the four mechanisms. Mechanism scores for each participant were created by taking their average score in each mechanism and dividing by the number of valid responses, which stopped data from being skewed where respondents stated a question was not applicable. Average mechanism scores were reversed (except of the item representing “relational integration”, which was phrased negatively), so that higher scores indicated greater perceived normalization. Higher scores represent better perceived implementation in relation to each mechanism. A Wilcoxon signed-rank test was done to check if two sets of related data are different from each other. These two sets were the variables “Do you consider Endre to be an integral part of your current day-to-day work?” and “Do you consider that Endre will become an integral part of your day-to-day work?”. A significant difference between the two items was seen as indicative that the use of Endre might become a more normal part of professionals’ work in the future.

#### 2.7.3 Triangulation

Data were triangulated by exploring (dis)agreements and silences across the qualitative and survey data sets. This was conducted initially by a single researcher (SP) identifying and listing subconstructs that demonstrated particularly high or low normalization, comparing these against qualitative themes and then discussed among the research team. Revisions and amendments to the qualitative themes were made based on the discussions with the whole team.

## 3 Results

### 3.1 The COVID-19 pandemic

The seminar series and the data collection started with the first session in clinics 1 and 2 (28th February and 2nd March 2020). Further sessions were cancelled by TGL 11th March 2020 due to the Covid-19 pandemic. The study was restarted early September with the second session in clinics 1 and 2 (4th and 7th September), and these clinics completed the seminar series without further delays (last session 30th October and 2nd November). Clinics 3 and 4 started 28th September and 27th October 2020 and completed 23rd March and 11th May 2021. These two clinics experienced multiple reschedules due to changing covid regulations. All sessions apart from the first session for clinics 1 and 2 were changed from physical to digital presentations before restarting in September 2020, when we realized that the pandemic might continue for a long time.

### 3.2 Question 1: what was the uptake of the digital alcohol intervention?

#### 3.2.1 Actual usage of the digital intervention and recruitment source

Forty patients registered their contact info in the system but six of these did not complete the registration process. To complete registration they had to open their e-mail inbox and activate a hyperlink unique to the user and then enter a pin code that they received on their phone (SMS). This means that incomplete registration may be due to registering an incorrect e-mail address or phone number. It can also be due to technical issues—we know that during a specific period the system failed to send SMSs to users. Of the 34 patients completing registration, nine completed less than six modules, nine completed six through 15 modules, and 16 completed 15 through 22 modules (10 completed all modules assigned to them). Even though about a quarter had an early dropout, almost half of patients completing the registration used the intervention as intended. Due to several technical problems during the study period, together with feedback from patients and staff, we have reason to believe that usage was somewhat lower than what could be expected without the technical problems.

The 34 patients that completed registration were recruited by 17 GPs, meaning that a little more than half of the GPs did not recruit any patient (*n* = 37 GPs). Note that recruited is not the same as referred—more patients were referred to the intervention than were actually enrolled in treatment.

#### 3.2.2 Inclusion and dropout survey

Of the 37 GPs at the four centers included, 16 responded to the inclusion and dropout survey (see Table 1). The responders estimated that they had recruited 57 patients in total (i.e., three reported recruiting none; nine reported one to five, and four reported six to ten patients). The database of the digital intervention contained only 25 patients that reported one of these 16 GPs as their GP. This means that the GPs overestimate how many patients they have recruited. Note that technical difficulties with the system probably have contributed to this discrepancy.

**Table 1:** Patients’ reasons for rejecting participation in the study/Endre.

Reason to reject participation	Number of responses
Disagree the alcohol is part of the problem	26
Do not want to participate in research	5
Do not have pc/pad/smartphone	5
Do not want to use a digital solution	9
Do not want to use Endre	18
Do not know/other reasons	1

GPs were also asked to provide the reasons patients gave for rejecting to participate in the study/Endre (see Table 1). The most frequent reason given was that the patient did not perceive alcohol to be part of the problem.

### 3.3 Question 2: what factors inhibited the implementation of the digital alcohol intervention?

#### 3.3.1 NoMAD survey and group interviews

The NoMAD survey was administered to all GPs and staff present at the seminar session, and all completed the survey (37 at baseline (after seminar 2 or 3) and 28 at follow up (after seminar 4)). Seven semi-structured group interviews with 17 staff members (GPs and support staff separately), were conducted (2–3 participants per interview). Three of the study sites participated in two group interviews (GPs and support staff) and one site participated in one group interview (GPs only). See Table 2 for a summary of interview participants and survey respondent characteristics.

**Table 2:** Participant characteristics of group interviews and surveys.

Characteristics	Survey respondents *N*		Interview participants *N*
How many years of experience do you have?	Before (total *n* = 37)	After (total *n* = 27)	(Total *n* = 17)
<1 year	2		0
1–3 years	10		1
4–10 years	15		3
>10 years	10		13
Professional role			
GP—own practice	20	14	9
Locum GP/specialist doctor/intern or GP in training	4	4	1
Medical secretary/nurse/other	13	9	7

#### 3.3.2 Perceived confidence and normalization

At the end of the seminar series participants reported that they were somewhat confident about their use of Endre (mean = 4.93, SD = 2.73), but that they did not consider Endre being an integral part of their current day to day work (mean = 3.85, SD = 2.27) (see Table 3). However, it was reported that Endre was somewhat likely to become a more integral part of their work (mean = 5.56, SD = 2.56), with a Wilcoxon signed-rank test confirming the difference was statistically significant (*p* < 0.001) suggesting that Endre was not yet fully embedded.

**Table 3:** Confidence and perceived normalization at the start and end of the training.

	Mean (SD) before	Mean (SD) after
How confident are you about your use of Endre?	4,66 (2.58)	4.93 (2.73)
Do you consider Endre to be an integral part of your current day-to-day work?	3.86 (2.25)	3.85 (2.27)
Do you consider that Endre will become an integral part of your day-to-day work?	6.34 (1.95)	5.56 (2.56)

Rated on a 10-point Likert scale with higher scores indicating greater confidence/normalization.

#### 3.3.3 NPT mechanisms and subconstructs

**Figure 1:**
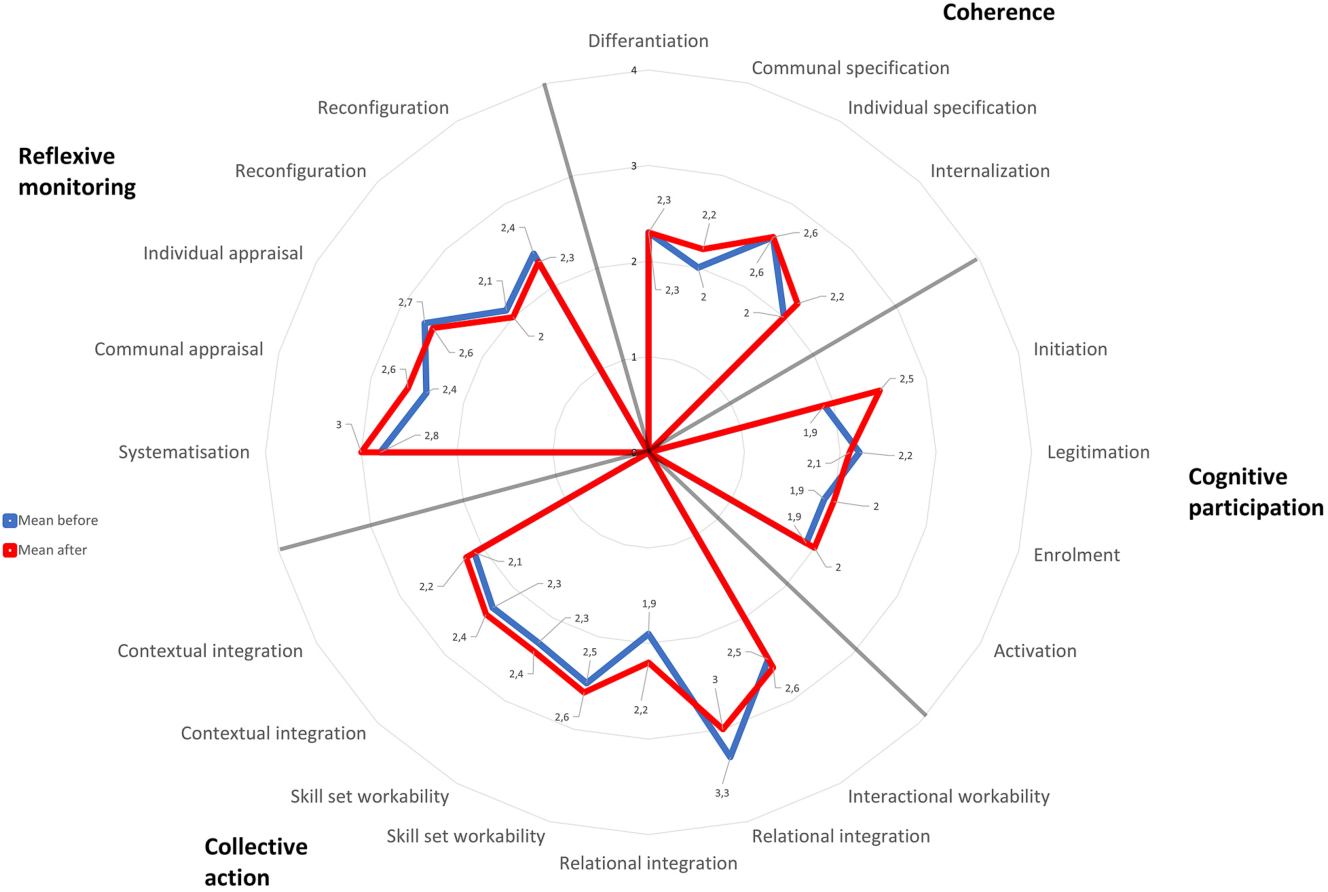
Petal chart showing mean scores for the 16 NPT subconstructs. Rated on a 5-point scale with higher values representing higher levels of normalization.

Descriptive analysis of the mean scores of the four NPT mechanisms indicated little to no improvement in the efforts to implement Endre. Overall, mean scores across all four constructs were low, ranging from 1.99 to 2.48 (scale 1–5; Table 4). Further analysis of the 16 subconstructs (Figure 1) showed that most subconstructs are indicative of unsuccessful implementation. Survey findings agreed with the qualitative findings. Supporting qualitative data (quotes) for the four main NPT mechanisms are provided in Table 5. The following section will focus on the key barriers identified in the qualitative analysis. The four identified barriers were triangulated with all the data sources and mapped onto the four main NPT mechanisms. Mean follow-up scores for the four NPT mechanisms are reported with each of the qualitative themes to aid interpretation.

**Table 4:** Descriptive statistics and reliability scores for the NoMAD at the start and end of the training.

Scale	Mean (SD) before	Mean (SD) after	Scale reliability (*α*)	Number of scale items
Coherence	2.25 (0.73)	2.31 (0.65)	0.48	4
Cognitive participation	1.99 (0.64)	2.15 (0.68)	0.50	4
Collective action	2.40 (0.83)	2.49 (0.77)	0.64	7
Reflexive monitoring	2.47 (0.66)	2.48 (0.73)	0.63	5

Rated on a 5-point scale with higher values representing higher levels of normalization.

**Table 5:** Summary of qualitative framework analysis for 4 NPT constructs with illustrative quotes.

NPT construct	Mapped theme	Illustrative quotes
**Coherence:** How do people work together to understand and plan the activities that need to be accomplished to put an intervention and its components into practice? *Mean survey score: 2.31*	**Need for tailored implementation support:** GPs felt like the seminar series did not adequately support them with delivering the therapeutic aspects of Endre. They thought the seminar series was overly focused on the technical aspects of delivering the intervention (as opposed to the therapeutic elements). Support staff on the other hand welcomed the opportunity to be involved in supporting the technical aspects of delivering the intervention. However, they did not feel like it was their role to guide patients in the behaviour change aspects of the intervention.	“*I thought that was maybe the less interesting and less needed part, thought it was repetitive from time to time, that they ran through the same things, and I think maybe we could've had one introduction of that, but then since we're not the ones who've been using it, but our secretaries, maybe we should just gone into two groups there.*” (GP, clinic 1) “*It must be that we feel we haven't had a chance to use it enough. We should really try it ourselves, so we know what we're passing on. The more we used it the more we'd have a chance to reflect over it.*” (Staff, clinic 3)
**Cognitive participation:** How do people work together to create networks of participation and communities of practice around interventions and their components? *Mean survey score: 2.15*	**Minimal involvement:** GPs and staff had a shared understanding of their role in the delivery of Endre. However, both perceived their role to be minimal and only supporting the procedural elements of intervention delivery. Therefore, there was a clear misalignment between how the intervention was supposed to be delivered (blended with GP taking an active role in guiding Endre use) and how it was delivered in practice (passive where GPs refer eligible patients and staff support technical sign up).	“*And there's a point there, to make it sure it's not uncomfortable to register. Yes. It good to be humble and open in that situation and not dig around and ask questions.*” (Staff, clinic 1) “*For the doctors it's getting these patients to start Endre. When they then get sent to us, we only start the computer, find Endre and leave them to it. That's it. You’re no more involved than that. We don't see anything they do or talk with them as you would when you're involved in stuff like that*.” (Staff, clinic 3)
**Collective action:** How do people work together to enact interventions and their components? *Mean survey score: 2.49*	**Diminished trust:** A range of factors diminished some GPs' trust in Endre, including technical issues, fear of labelling patients and lack of patient adherence. In some of the sites this led to a lack of enrolment and continued support for the intervention. Some GPs did not support the intervention out of their concerns around the long-term availability of the intervention.	“*We were informed after the fact, because there were technical issues with the passwords. They didn't receive the password they were waiting for. We sent them away before they received it, but they've sorted it now*.” (Staff, clinic 3) “*Many people were afraid they'd be labelled as someone with an alcohol problem if they participate. (.) In that sense I feel the name Endre (Change) is wrong. So, I think it could be named something more benign instead*” (GP, clinic 4)
**Reflexive monitoring:** How do people work together to appraise interventions and their components? *Mean survey score: 2.48*	**Missing feedback loop and follow-up:** GPs and staff felt that there was a missing feedback loop regarding patients' use and impact of Endre. GPs would have liked to receive feedback on the implementation (i.e., number of registrations and completions) of Endre to help monitor engagement. They would have also liked to receive summaries of patients’ progress in Endre that they could use in follow-up appointments. Both GPs and staff would have liked to receive patient testimonials to help them tailor their use of the intervention.	“*I'd like feedback regarding how you follow up on the patients who have started Endre. Perhaps I had them back too infrequently, but I felt I was leaving them to go through a program with a psychologist or someone in Endre. Where they would get help.*” (GP, clinic 3) “*I'd like to be informed when the patient has been using Endre. Then I have something to work from in the next consultation or in a consultation I can plan based on the patients answers and what has come from Endre.*” (GP, clinic 3)

##### 3.3.3.1 Coherence: need for tailored implementation support

Staff felt that the training did not adequately support them in planning and understanding the implementation of Endre (survey score 2.31). Even though Endre training was part of each of the four sessions in the clinical seminar series, staff perceived this training to be repetitive and not tailored to their individual needs. GPs thought that the training should have focused exclusively on the support staff given their role in supporting the technical aspects of the intervention.

“I thought it was repetitive from time to time, that they ran through the same things [Endre]. Since we're [GPs] not the ones who’ve been using it, but our secretaries, maybe we should have just gone into two groups.” (GP 1, clinic 2)

Similarly, some GPs thought that the seminars involved a lot of clinical information on alcohol related health problems, which was perceived to be less relevant for their support staff.

“The technical details which the health secretaries are a part of was directed to them I thought, but the other information was more for us. So, I think they could join us one hour.” (GP 1, clinic 3)

Both GPs and support staff reported that they felt like the Endre training was overly focused on the technical aspects (e.g., sign-up and troubleshooting) rather than supporting the therapeutical process on how to guide patients’ use of the intervention.

“There should have been more room in the course as well because it came, it was a bit fast and quick at the end of every day, a little session about Endre. So, I think it took time and if we actually managed to find out how it worked, we got access to a test user at the end. But it would have been good to have it in place earlier and that we could also…we would have liked to practice using Endre together. Because it's, it's a bit unclear how many are actually comfortable with how it actually works. So, either get a presentation, spend more time introducing Endre, or use a little time on the course to practice using it.” (GP 2, clinic 1)

Many mentioned that they would have welcomed the opportunity to try Endre as part of the training, so that they would feel more comfortable in supporting patients in their use of the intervention.

“We should really try it ourselves, so we know what we're passing on.” (Staff 2, clinic 3)

At the end of the last clinical seminar GPs and support staff reported minimal to no use of Endre in their routine practice. The lack of GP engagement with the intervention was due to some of the other observed barriers, including their perceived (passive) role in the delivery, the missing feedback loop, and the diminished trust in Endre. Other reasons perceived to disable the engagement with Endre included limited patient interest, lack of user adherence, and lack of time to discuss Endre.

“Some of the patients I had they… eh… they were very interested in Endre while still in my office, but then they didn't register afterwards. Which is fine. And then there were a couple who eh… registered and used it where we scheduled eh… check-ups and we were planning on using Endre as a part of the… the evaluation, but they quit” (GP 2, clinic 4)

##### 3.3.3.2 Cognitive participation: minimal involvement in intervention delivery

There was a lack of overall participation in the implementation of Endre (survey score 2.15). Even though Endre was designed as a blended intervention that could be used to facilitate the clinical consultation, most GPs did not think that they were meant to actively guide patients’ use of Endre. At the end of the clinical seminar series many GPs were still unsure about their role in delivering Endre.

“I think that should have been made clear, that you as doctors should do this and that. I didn't understand it like that at all. I felt it was more of a survey which then would go through the patient and those involved in Endre.” (GP 1, clinic 3)

Consequently, GPs and support staff perceived their role in delivering Endre as minimal and passive. GPs perceived their role to be limited to referring eligible patients to their support staff who would then help the patients with signing up for the intervention.

“In consultation I don't have the time to actually go in on the app, so we organized it so that they could start up with the secretary afterwards.” (GP1, clinic 2)

Support staff welcomed the opportunity to be involved in the delivery of Endre, but saw their role limited to supporting the technical aspects of intervention delivery.

“I thought that it was exciting to be able to be a bit involved with it, yes. And I would like to continue to work with it and have some follow-up meetings. (Staff 1, clinic 1)

“So, you’re incredibly uninvolved. Only opening the computer, pressing Endre and no more.” (Staff 1, clinic 3)

From the perspective of support staff, alcohol is a sensitive topic, and it was not “*their responsibility to discuss that with patients*” (Staff 1, clinic 3). They acknowledged that these patients may be vulnerable and that it would therefore be best to “*be polite and respectful to them*” (Staff 2, clinic 2). From their perspective it was the GPs’ role to guide the clinical aspects of delivering Endre.

##### 3.3.3.3 Collective action: diminished trust in intervention

Technical issues at one of the study sites diminished GPs’ trust in and engagement with Endre (survey score 2.49). Patients who signed up for Endre at this site did not receive a confirmation email with the password to log into their account.

“The spark ignited at the start in engagement with Endre fizzled out due to the number of bugs. We felt it came too early in the stage of the process. This was almost at the development phase. We can't say we will continue to use this, we don't think, yes there's the solution for us.” [GP 1, clinic 3]

Some GPs also reported receiving negative feedback from patients with regards to the framing of questions asked in Endre. According to their patients, some of the questions were framed in a way that it labelled the user as someone with an alcohol problem. Consequently, some GPs felt uncomfortable referring their patients to Endre out of concerns of labelling them as “alcoholics”.

“One of them found it too stressful and the other one thought some of the questions were a bit, eh…how should I say it? Eh… yeah… he was given the impression that he was an alcoholic, due to the phrasing of the questions. And he didn't like that because that's not how he thinks of himself.” (GP 2, clinic 4)

One of the GPs reported their concerns regarding the long-term availability of Endre, which may have further contributed to the limited trust in the intervention. This GP highlighted the importance of referring patients to an intervention that they knew they could rely on going forward.

“I don't know if my patients will have access to Endre after that. So, that's why I'm currently thinking that this is something my patients can have access to by joining the research project (but) that it's time limited. It could be available afterwards, I don't know, but… so I didn't mean anything negative about using Endre, but rather (looking for) something that I know I can use over time.” (GP 3, clinic 1)

##### 3.3.3.4 Reflexive monitoring: missing feedback loop and follow-up

GPs felt that there was a missing feedback loop regarding patients’ use of Endre and the impact that it had on their alcohol related health problems (survey score 2.48). Regarding the use of Endre, GPs would have liked to receive information on how many patients registered and completed the intervention. GPs would also have appreciated a summary of their patients’ progress within Endre, that they could use in a follow-up appointment.

“I'd like to be informed when the patient has been using Endre. Then I have something to work from in the next consultation or in a consultation I can plan based on the patients answers and what has come from Endre.” (GP 1, clinic 3)

GPs reported that some of their patients also requested more active guidance in their use of Endre. Most of the GPs thought that there should be a standard timeframe (e.g., every 2 months) after which they would book a follow-up appointment to discuss patients’ progress and any difficulties that they may have. To support this, some GPs would have “*liked feedback regarding how you follow-up on the patients who have started Endre*” (GP 1, clinic 3). Given the lack of systematic feedback, some GPs opted to refer their patients to specialized services that did provide a feedback loop.

“And since I also haven't had any feedback about Endre I will likely refer them to a conversation at the addiction centre instead, if I thought that was necessary. Because then I'd also get feedback on how it went and what they talked about.” (GP 2, clinic 3)

Lastly, some GPs and support staff members mentioned that they would have liked to read about patient and professional testimonies. They thought that hearing about positive case examples would have increased their trust in Endre and motivated their continued use of the intervention.

“To hear some feedback from patients, it's not necessarily our patients so we don't hear anything. It could be from other centres too.” [Staff 2, clinic 3]

“Yes, exactly with these last points. That would make us more engaged.” [Staff 1, clinic 3]

## 4 Discussion

Digital alcohol interventions delivered in primary care can help reduce alcohol consumption, but their widespread implementation is lagging behind. The current mixed-methods feasibility study aimed to investigate the uptake, barriers and facilitators to the implementation of a digital alcohol intervention for at-risk drinkers in Norwegian primary care. The findings suggest that the digital alcohol intervention (Endre) did not become fully embedded across the four study sites. Given the rising demand for clinically- and cost-effective solutions for tackling increased alcohol consumption, these findings suggest that more consideration needs to be given to how we can effectively embed such interventions into routine clinical practice.

Support staff in this study perceived the training in the digital intervention to be generic and not tailored to their needs. They also thought that there was too much focus on the procedural, rather than the therapeutic elements of intervention delivery. This highlights the importance of implementation strategies that are tailored to the needs of service providers (45). The clinical seminar series was primarily designed to address GPs’ needs for relevance-based clinical strategies for addressing alcohol in primary care. This involved tailored training in a range of clinical strategies for addressing alcohol and related health problems in the clinical consultation (e.g., training in motivational interviewing). Endre was included in the seminars based on GPs’ need for a digital resource for patients’ use between sessions. However, given the complexity of incorporating a digital intervention into the practice workflow, there is a need for involving stakeholders more systematically in the design and implementation planning of the intervention (46). Such an approach should focus on maximizing compatibility with professional workflows and needs.

Another challenge to the implementation of Endre was the passive involvement of support staff in the therapeutic process. Although Endre was designed to be delivered as a blended intervention, wherein GPs were meant to guide patients’ use of the intervention, this was not communicated clearly enough during the clinical seminar series. Future implementation initiatives should focus on enrolling staff to be actively involved in guiding patients’ use of digital interventions in primary care. Previous research identified that patients would like staff to be “invested” in the delivery of digital alcohol interventions by holding patients accountable for their behavior change (18). However, this also points to a dilemma, between minimizing extra workload for the GP, and improving the patient's follow-up by involving the GP more. The current resource crisis and increased workload in primary care in several countries is an important background. A well-functioning digital tool should ideally improve follow-up for the patient and support the patient-doctor relationship without increasing the workload for the GPs, e.g., by assisting the patient in preparing for follow-up consultation, and providing important information and concerns directly to the electronic patient record for the GP (47).

Some participants said they wanted to try Endre prior to patient recruitment. This opportunity was offered to all clinics via email initially, but we did not focus on this in the sessions. It is very likely that allocating time in the sessions to explore Endre themselves and helping them sort out problems and get to know Endre better, would improve their engagement and competence. In addition, monitoring how Endre was used by the participants in this initial phase would have allowed us to offer more information and support to those not trying out Endre themselves. Transforming the sessions to digital format without extra resources added to this problem. The participants’ needs were therefore not properly acknowledged by the organizers in this setting, and thus not met.

The minimal involvement of GPs was also related to their inability to monitor the usage and impact of Endre. This was reflected in GPs’ overestimation of the number of patients that they recruited. GPs would have appreciated periodical summary reports of the number of Endre registrations and completion rates. Such information may be particularly helpful when evaluating and improving the implementation of a digital solution like Endre [e.g., using rapid cycles of improvement (48)]. Regarding the impact of Endre, GPs would have liked to receive summary reports on patients’ behavior change progress, which they could use to further support the patients in the next consultation. This raises some potential issues with regards to patients’ concerns around privacy (49). Findings from our separate qualitative study with patients indicate that they liked the fact that they could fully confide information about their alcohol consumption to Endre, without having to worry about being judged by a real person. An alternative may be an added function that allows patients to send a summary report to their clinician if they choose to do so. Another option could be to integrate a function that would allow patients to make a list of topics relating to their health problems or alcohol consumption that they would like to discuss with their GP. This could help patients to prepare for the consultation and allow them to have a more informed conversation with their GP.

Our findings demonstrated that support staff welcomed the opportunity to be involved in delivering Endre. Thus, there may be some scope to increase their involvement beyond supporting the registration on the intervention and letting them support patients with their behavior change. This finding echoes a UK study, which demonstrated GPs’ mixed views on “role legitimacy” (perceived boundaries of the right to intervene) when it comes to alcohol screening and intervention (20). GPs in that study thought it was more appropriate, and realistic, for nurses to be more actively involved in the delivery of such preventive interventions. However, currently in Norway there are some barriers to increased involvement of support staff in clinical procedures. Most GPs in Norway are self-employed, and their income consist of (1) basic financial support dependent on the number of registered patients, (2) patient payment [or reimbursement for children or when exceeding 3000 NOK (2022, approx. 260 EUR) in patient charges, included medication for chronic diseases per year], and (3) reimbursement for specific procedures or extra time spent. The majority of patient-paid or reimbursed procedures require that they are performed by the GP, significantly limiting the potential for transferring e.g., diagnostic procedures or follow-up to support staff. The categories of and number of extra staff in Norwegian GP clinics are therefore limited compared to in UK and other countries (50). A governmental pilot project (completed March 2023) was aimed at increasing interdisciplinary teamwork in primary care clinics, but patient payment or reimbursement for procedures performed by other than the GP is still restricted.

GPs in this study reported diminished trust in Endre over time, which further prevented the digital intervention from becoming embedded in practice. Some of the trust was diminished due to a technical error in one of the implementation sites preventing patients from being able to create a user account. This highlights the need to ensure that there are no technical issues prior to the main roll-out of a digital intervention, and that incidents occurring after roll-out are swiftly recognized and managed. One way this could be achieved is through the embedding of digital interventions on larger integrated digital health platforms (51). Implementation embedded in a national integrated digital platform would also facilitate ongoing funding for and long-term availability of the intervention.

One of the strengths of this study was the application of the Normalization Process Theory (NPT) as an analytical framework for understanding barriers and facilitators to the implementation of Endre. We applied NPT both in the qualitative (as a coding framework) and the quantitative (using the NoMAD survey) components of the study. To our knowledge this is the first study which translated the NoMAD survey into Norwegian using a recommended method for translating research instruments (42). The low overall normalization score (3.85 out of 10) clearly demonstrated that Endre did not become fully embedded by the end of the feasibility study. Nonetheless, it is worth noting that participants felt that Endre may become an integral part of their work in the future (indicated by a score of 5.56 out of 10). We did not observe a meaningful change in the low mean scores across the 16 NPT subconstruct from baseline to follow-up. One reason for this could be the limited opportunities that support staff had to use Endre, which was further complicated during the outbreak of the Covid-19 pandemic. Another reason could be the high rate of patients who declined to use Endre, mainly due to their perceived lack of relevance to their health problem. This highlights the need for more comprehensive training for professionals in communicating the relevance of alcohol to their patients’ health problems (25).

2Triangulating the quantitative findings with the qualitative themes provided additional insights into why the implementation of Endre remained challenging. The mapping of themes made it clear that additional work was needed across all four NPT mechanisms, including coherence (how people make sense of the intervention), cognitive participation (how people get involved in the intervention), collective action (how people work together to make the intervention work), and reflexive monitoring (how people assess the impact of the intervention). With regards to the relationships between NPT mechanisms we observed them to function in a dynamic, non-linear way. For example, GPs’ inability to assess information on the uptake and impact of Endre (reflexive monitoring) meant that they felt less invested (cognitive participation), which undermined their confidence in enacting the intervention (collective action). Equally, technical issues (collective action) led to decreased scope for participation by professionals and patients (cognitive participation). Based on our findings we provide several recommendations for implementing digital alcohol interventions in primary care (see Table 6).

**Table 6:** Recommendations for improving implementation of digital alcohol interventions in primary healthcare settings.

Coherence: need for tailored implementation support	Cognitive participation: need for active stakeholder involvement
• Involve professionals and patients in the development of the digital tool to facilitate interactional and skill set workability. • Tailor implementation strategies that address the needs of professionals delivering and patients receiving the intervention.	• Enrol staff to be actively involved in guiding patients’ use of digital interventions in primary care. • Build and sustain a community of practice around the delivery of the digital intervention.
Collective action: need for contextual integration	Reflexive monitoring: need for intervention feedback systems
• Fix technical issues and provide ongoing technical support. • Integrate the digital tool in existing digital health platforms to facilitate maintenance.	• Provide professionals with summary reports on patient progress to enable personalized support. • Provide professionals with data on the number of registrations and completions.

### 4.1 Limitations

One limitation is the small sample size for the quantitative component of the study. This means that the before-and-after measure on the NoMAD questionnaire was not powered to demonstrate statistical differences. Due to several complications in the study delivery, we did not collect identifiers for NoMAD respondents, which means that we could not distinguish whether those responding to the follow-up survey were the same participants as at baseline. However, given that NoMAD focuses on team-based processes of implementation it is reasonable to assume that the measure would detect change on a practice level. Furthermore, having the qualitative data helped us interpret the NoMAD results and get a better understanding of implementation status in this study. Another limitation was that GPs were asked to respond to the inclusion and drop out survey from memory at the end of the study, which could have introduced some recall bias. All training sessions, apart from the first session for the first two sites, had to be changed from physical attendance to digital presentation. The implementation of Endre was further complicated by several regulations and restrictions during the pandemic. The regular activity and the number of patients allowed in the clinic at the same time were significantly reduced, and many patients received digital consultations. Various infection control procedures affected the process of signing up to Endre, and for longer periods the clinics were assigned as vaccination centers for their old patients and patients with chronic medical problems. Consequently, the time and attention available to assist patients signing up to Endre was significantly reduced. Lastly, this study is limited to the views of healthcare staff and does not report on the perspectives of patients. Patient interviews were conducted and will be reported in a separate article.

## 5 Conclusions

Implementing and normalizing digital innovations in primary care requires primary care delivery teams to work together to incorporate new workflows into their clinical routines. This study suggests that primary care staff would like to take an active role in guiding and personalizing patients’ use of digital solutions, but that they require tailored support and feedback systems to facilitate this work. To ensure that support meets staff needs, it is important to actively involve them in the development and implementation of new digital innovations. Digital tools need to be designed to facilitate ease of use by both patients and professionals. Integration of digital solutions may further be improved by including features such as dashboards that enable clinicians to access and monitor patient progress and self-reported outcomes. Future research will be needed to evaluate whether tailored implementation strategies lead to improved normalization and uptake of digital alcohol interventions.

## Data Availability

The datasets presented in this article are not readily available because this would likely compromise participants’ anonymity. Some descriptive data may be available from the corresponding author on reasonable request. Requests to access the datasets should be directed to torgeir.gilje.lid@sus.no.
