# Supplementary material for: Recommendations for implementing digital alcohol interventions in primary care: lessons learned from a Norwegian feasibility study
Source: Front Health Serv. 2024 Oct 11;4:1343568. doi: 10.3389/frhs.2024.1343568 (PMC11514074; doi:10.3389/frhs.2024.1343568)
Supplement: Supplementary file 1 [file Supplementaryfile1.docx]

Supplementary Material

**Supplementary Figures and Tables**


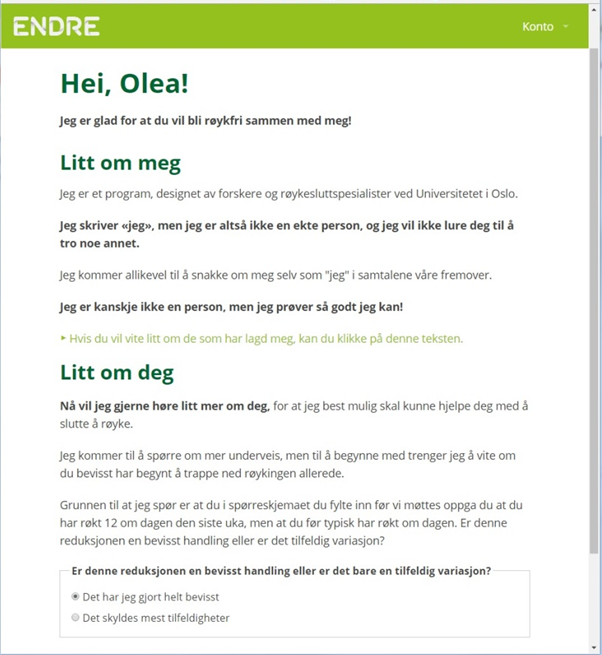


**Supplementary Figure 1.** Screenshot 1 of the Endre e-health intervention


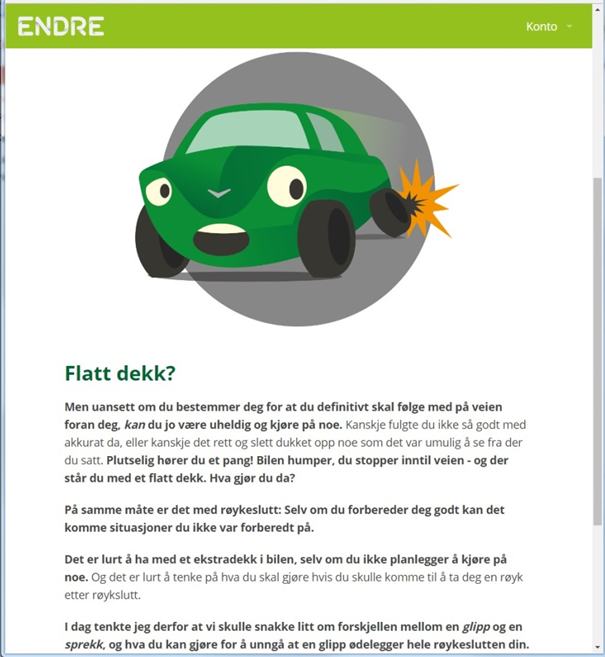


**Supplementary Figure 2.** Screenshot 2 of the Endre e-health intervention

**Supplementary Files 1. Topic guide for GP interviews**

**Introduction**

- Introduce yourself
- Introduce the purpose of the interview
  - To understand their views on the clinical topic seminar series on alcohol and related health problems
- Inform about: Audio recording, anonymisation, stopping interview
- Reaffirm participant consent

**Background information (to be collected prior to the interview)**

- What is your role in the group practice? (will collect this data ahead of interview)
- How long have you been working as a GP? (will collect this data ahead of interview)

**General questions**

- What was your overall impression of the clinical topic seminar series?
  - Any likes / dislikes
- What did you think of the format of delivery?
  - Number / duration of the sessions
  - Group format including all members of staff
  - Virtual delivery of certain components
  - Homework assignments
- Was there anything that facilitated or hindered your learning experience?

**Pragmatic case finding questions**

- How relevant was the topic (alcohol) for your own practice?
- To what extent did the seminars help you think about alcohol-related health problems in a different way?
- Did you learn more about the different medical conditions in which alcohol may be relevant?
- To what extent do you feel you can use this new knowledge to support your patients with changing their alcohol habits / helping the patient improve their health?

**COM-B questions**

- Do you feel like you have the necessary time and resources to apply the new knowledge / skills in your own practice? If not, are there certain elements of the training that you will use?
- How motivated are you to apply the knowledge / skills that you have learned in your own practice?
  - Anything that makes you less / more motivated?
- Have the seminars helped you develop a habit around addressing alcohol with your patients?
  - How long did it take you to form a routine around addressing alcohol?
  - If so, what helped making it a habit / what were the barriers to making it routine?
- How often do you consult patients who suffer from alcohol-related health problems?
  - Has there been a change in the number patients who you consult about alcohol?

**Views on specific seminar sessions (option)**

- ***Havar*** – introduction and support in using Endre
- ***Torgeir*** – alcohol related health problems; alcohol & aging; challenging situations (driver’s license, parenting, interaction with addictive medical substances e.g. sleeping pills, pain killers and tranquilizers)
- ***Sven*** – alcohol related health problems (interactions alcohol & health; treatment aspects, MI techniques)
- ***Aasa*** –motivation and change
- ***Expert discussions*** - nurse delivering community treatment & patient representative

**Final remarks**

- Any final comments?
- Thank you for your participation

**Supplementary Files 2. Topic guide for support staff interviews**

**Introduction**

- Introduce the purpose of the interview
  - To understand their views on the clinical topic seminar series on alcohol and related health problems
- Inform about: Audio recording, anonymisation, stopping interview
- Reaffirm participant consent

**General questions**

- What was your overall impression of the clinical topic seminar series?
  - Any likes / dislikes
- What did you think of the format of delivery?
  - Number / duration of sessions
  - Group format including all members of staff
  - Virtual delivery of certain components
  - Homework assignments
- Was there anything that facilitated or hindered your learning experience?

**Staff specific questions**

- How meaningful was it for you, as a member of staff, to be part of this topical seminar series?
- What parts of the seminars were most relevant for you?
  - Any changes to the seminar to make it more relevant (e.g., dealing with specific challenges)?
  - Separate part for staff only (e.g., dividing into groups)?
  - Any parts that could have been skipped for members of staff?
- Did you experience any change in how the clinic as a whole deals with alcohol and alcohol-related health problems?
  - Changes in dealing with patients
  - Changes in culture around addressing alcohol and related health problems

**Final remarks**

- Any final comments?
- Thank you for your participation

**Supplementary file 3.** Norwegian adapted Normalization MeAsure Development (NoMAD) survey

**Nomad spørreskjema**

### **Brukerveiledning**

**Dette spørreskjemaet er utviklet for å få en bedre forståelse av hvordan nye tiltak kan integreres i helsetjenesten.**

I spørreskjemaet spør vi om anvendelsen av *Endre*, et digitalt behandlingstiltak om alkoholvaner og helse for pasienter i allmennpraksis. Vi ønsker å undersøke hvordan Endre er integrert i det kliniske arbeidet ved legesenteret. Helsepersonell involvert i bruken kan være leger (fastleger, fastlegevikarer, ALIS eller LIS1) og ansatte (helsesekretærer, sykepleiere, osv) ved legesenteret.

Dette skjemaet har tre deler.

I del A stiller vi noen korte spørsmål om deg selv og din rolle.

I del B stiller vi tre generelle spørsmål om *Endre.*

I del C presenterer v konkrete utsagn om *Endre, som du må ta stilling til*.

For hvert av utsagnene i del C må du først vurdere om utsagnet er relevant for deg. Hvis utsagnet er relevant for deg, skal du vurdere i hvor stor grad du er enig eller uenig i utsagnet.


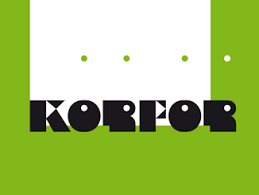


# Del A: Om deg selv

*Marker svaret ditt ved å sette et kryss i den grønne boksen.*

### 1: Hvor mange år har du arbeidet på legesenteret?

###
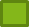

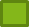
< 1 år 4-6 år > 10 år 1-3 år 7-10 år

### 2: hvilken yrkesgruppe tilhører du? fastlege – egen hjemmel eller fast ansatt Fastlegevikar/ALis/lis1 Helsesekretær/sykepleier etc

# del b: generelle spørsmål om endre

*Marker svaret ditt ved å sette et kryss på streken, eller en ring rundt ett av tallene.*

| Hvor fortrolig er du med å bruke *Endre?*   \| Føles uvant \|  \|  \|  \|  \|  \|  \|  \|  \| Helt fortrolig \| \| --- \| --- \| --- \| --- \| --- \| --- \| --- \| --- \| --- \| --- \| \| 1 \| 2 \| 3 \| 4 \| 5 \| 6 \| 7 \| 8 \| 9 \| 10 \| |
| --- | --- | --- | --- | --- | --- | --- | --- | --- | --- | --- | --- | --- | --- | --- | --- | --- | --- | --- | --- | --- |
| Føler du at *Endre for tiden er en naturlig del av ditt arbeid?*   \| Nei \|  \|  \|  \| Delvis \|  \|  \|  \|  \| Fullt og helt \| \| --- \| --- \| --- \| --- \| --- \| --- \| --- \| --- \| --- \| --- \| \| 1 \| 2 \| 3 \| 4 \| 5 \| 6 \| 7 \| 8 \| 9 \| 10 \| |
| Føler du at *Endre* vil bli en naturlig del av ditt arbeid*?*   \| Nei \|  \|  \|  \| Delvis \|  \|  \|  \|  \| Fullt og helt \| \| --- \| --- \| --- \| --- \| --- \| --- \| --- \| --- \| --- \| --- \| \| 1 \| 2 \| 3 \| 4 \| 5 \| 6 \| 7 \| 8 \| 9 \| 10 \| |

# del C: Utsagn om endre

*For hvert av utsagnene må du først vurdere om utsagnet er relevant for deg, og så vurdere i hvor stor grad du er enig eller uenig i utsagnet.*

|  | Veldig enig | Enig | Verken enig/ uenig | Uenig | Veldig uenig | Ikke relevant nå |
| --- | --- | --- | --- | --- | --- | --- |
| Jeg kan se hvordan Endre er forskjellig fra hvordan vi vanligvis jobber |  |  |  |  |  |  |
| På legesenteret har vi en felles forståelse av hva som er hensikten med Endre |  |  |  |  |  |  |
| Jeg forstår hvordan Endre påvirker måten jeg jobber på |  |  |  |  |  |  |
| Jeg ser den potensielle verdien av Endre for arbeidet mitt |  |  |  |  |  |  |

|  | Veldig enig | Enig | Verken enig/ uenig | Uenig | Veldig uenig | Ikke relevant nå |
| --- | --- | --- | --- | --- | --- | --- |
| Det er leger eller ansatte på legesenteret som følger opp at Endre tilbys aktuelle pasienter, og involverer alle på legesenteret |  |  |  |  |  |  |
| Jeg mener at det å gi Endre til pasienter er en naturlig del av min rolle |  |  |  |  |  |  |
| Jeg er åpen for å samarbeide med mine kolleger på nye måter ved å bruke Endre |  |  |  |  |  |  |
| Jeg kommer til å fortsette å støtte bruken av Endre |  |  |  |  |  |  |

# del C: Utsagn om endre

*For hvert av utsagnene må du først vurdere om utsagnet er relevant for deg, og så vurdere i hvor stor grad du er enig eller uenig i utsagnet.*

|  | Veldig uenig | Uenig | Verken enig/ uenig | Enig | Veldig enig | Ikke relevant nå |
| --- | --- | --- | --- | --- | --- | --- |
| Jeg kan lett integrere Endre i mitt vanlige arbeid |  |  |  |  |  |  |
| Endre forstyrrer arbeidsprosesser |  |  |  |  |  |  |
| Jeg har tiltro til at andre kan bruke Endre |  |  |  |  |  |  |
| De/den som har de nødvendige ferdighetene har fått ansvaret for å følge opp bruken av Endre |  |  |  |  |  |  |
| Det er gitt nok veiledning til å kunne bruke Endre |  |  |  |  |  |  |
| Det er nok ressurser tilgjengelig til å kunne bruke Endre |  |  |  |  |  |  |
| Legene på legekontoret støtter og legger til rette for å bruke Endre |  |  |  |  |  |  |

# del C: Utsagn om endre

*For hvert av utsagnene må du først vurdere om utsagnet er relevant for deg, og så vurdere i hvor stor grad du er enig eller uenig i utsagnet.*

|  | Veldig enig | Enig | Verken enig/ uenig | Uenig | Veldig uenig | Ikke relevant nå |
| --- | --- | --- | --- | --- | --- | --- |
| Jeg kjenner til rapporter og tilbakemeldinger om nytten av Endre |  |  |  |  |  |  |
| Leger og ansatte på legesenteret er enige om at Endre er verdt innsatsen |  |  |  |  |  |  |
| Jeg verdsetter effekten Endre har hatt på mitt arbeid |  |  |  |  |  |  |
| Tilbakemeldinger på bruken av Endre kan bidra til forbedre programmet i fremtiden |  |  |  |  |  |  |
| Jeg kan tilpasse hvordan jeg bruker Endre |  |  |  |  |  |  |

Tusen takk, du er nå ferdig med skjemaet!
